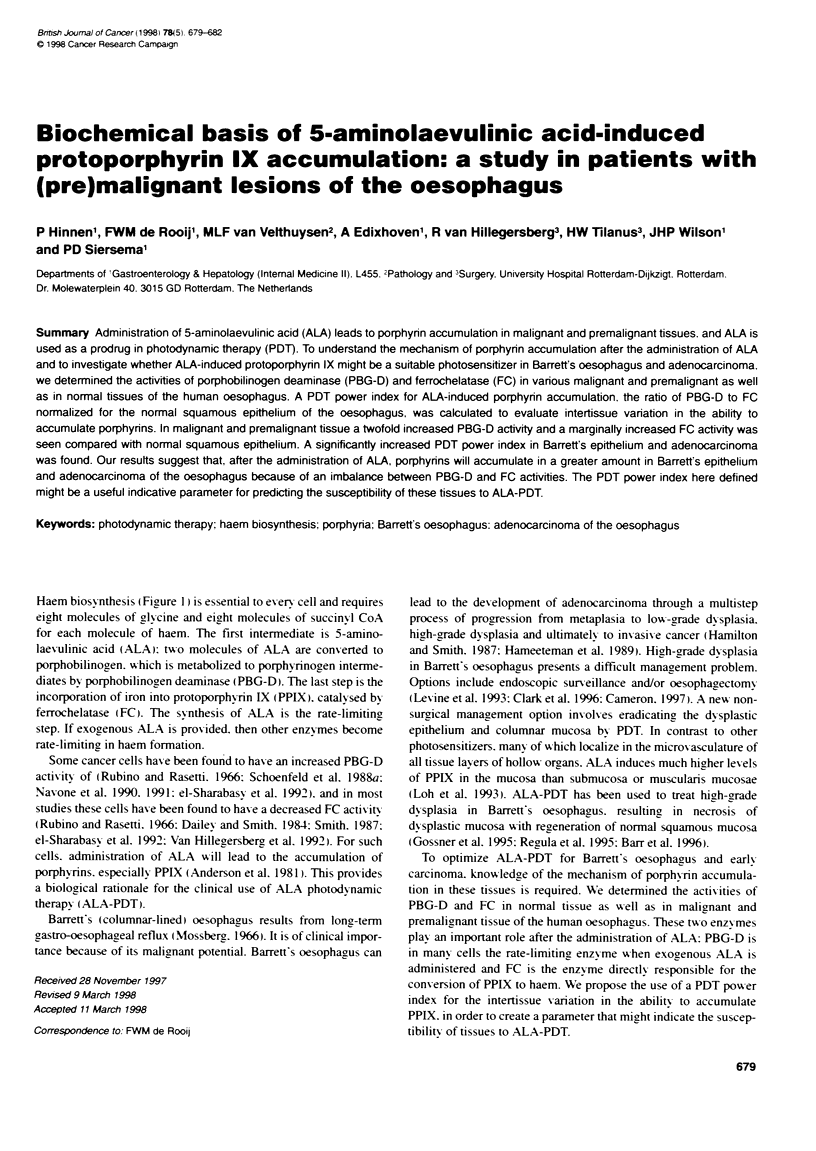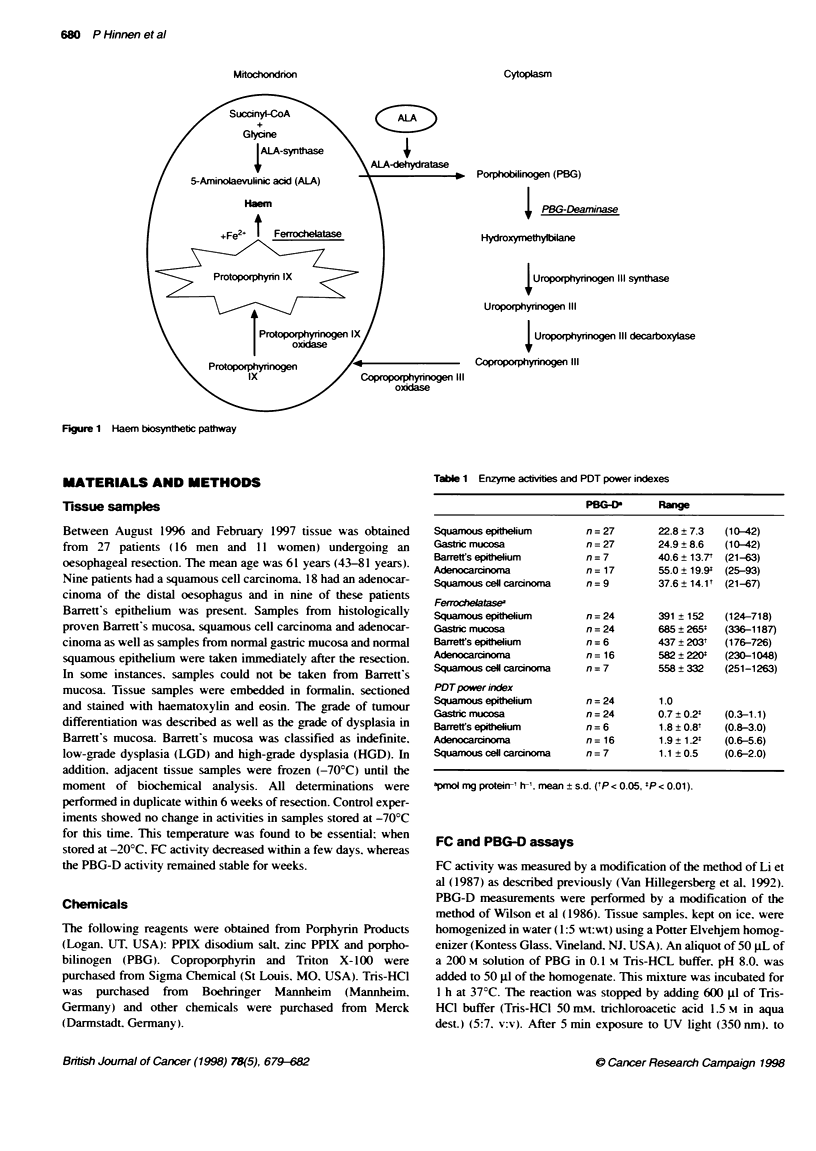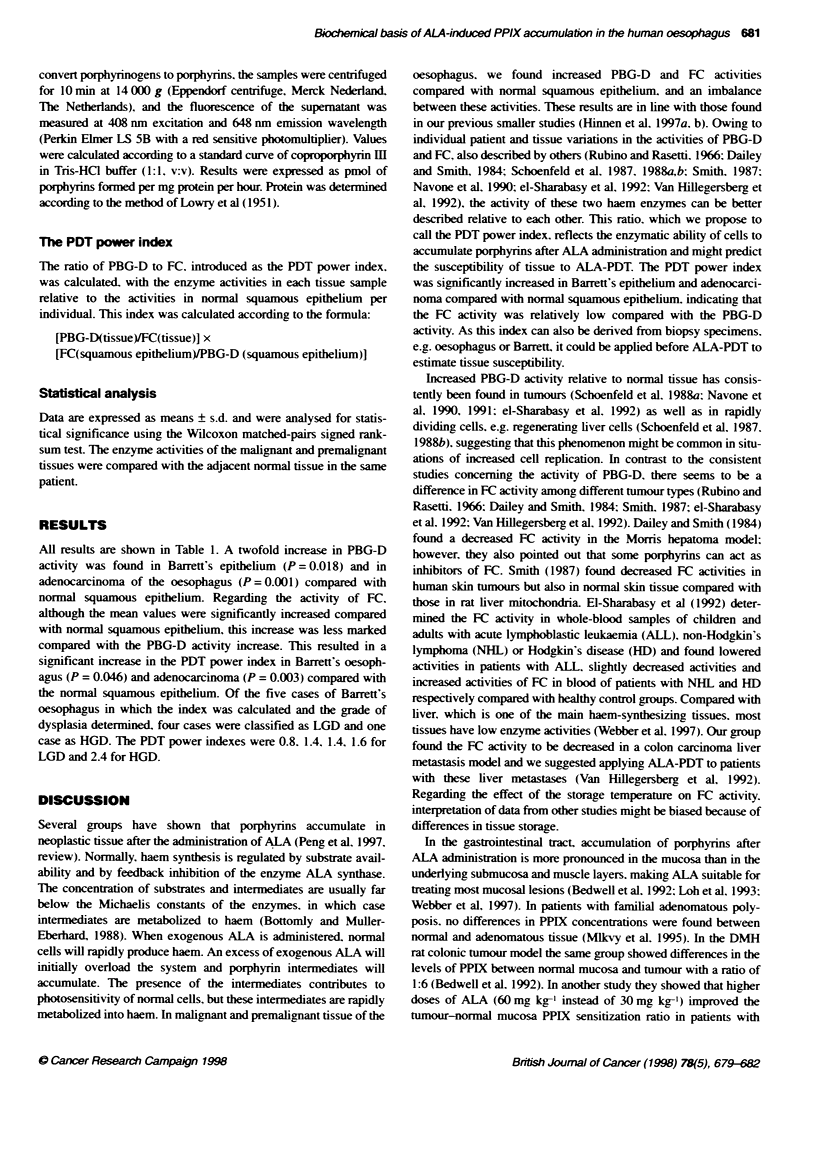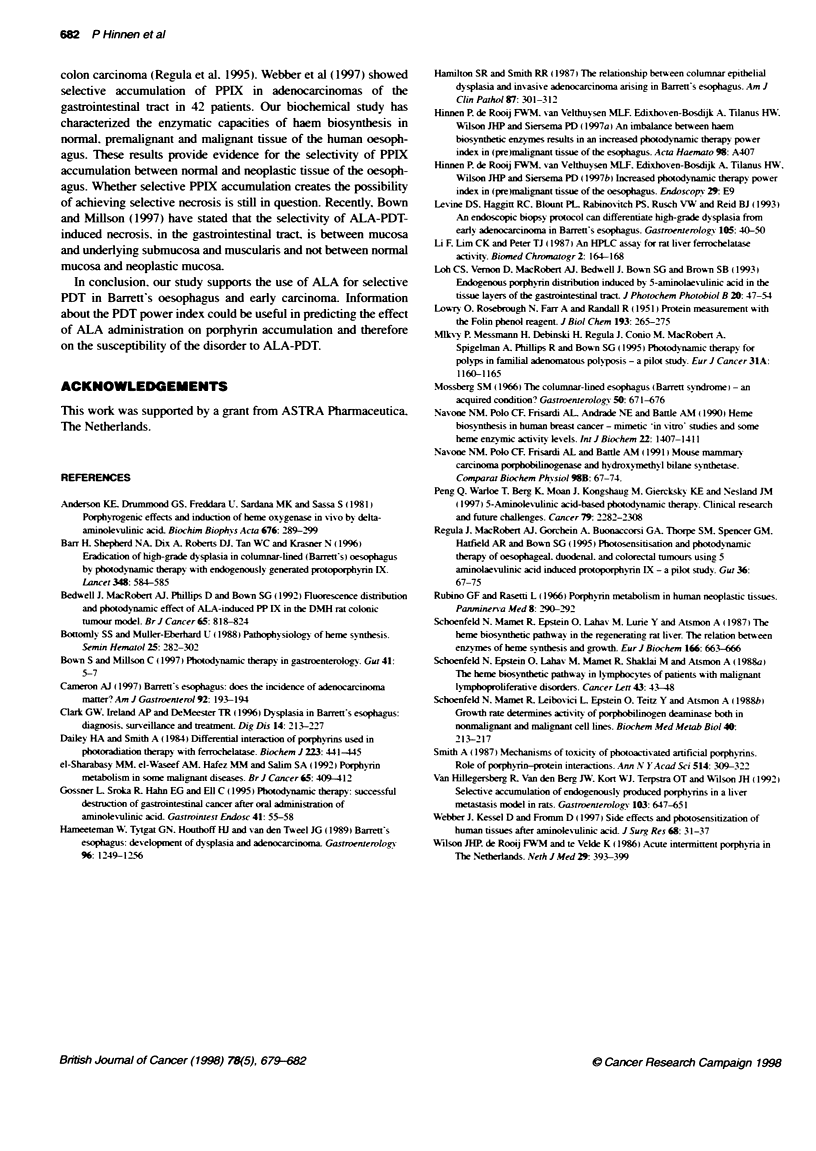# Biochemical basis of 5-aminolaevulinic acid-induced protoporphyrin IX accumulation: a study in patients with (pre)malignant lesions of the oesophagus.

**DOI:** 10.1038/bjc.1998.559

**Published:** 1998-09

**Authors:** P. Hinnen, F. W. de Rooij, M. L. van Velthuysen, A. Edixhoven, R. van Hillegersberg, H. W. Tilanus, J. H. Wilson, P. D. Siersema

**Affiliations:** Department of Gastroenterology & Hepatology (Internal Medicine II), University Hospital Rotterdam-Dijkzigt, The Netherlands.

## Abstract

Administration of 5-aminolaevulinic acid (ALA) leads to porphyrin accumulation in malignant and premalignant tissues, and ALA is used as a prodrug in photodynamic therapy (PDT). To understand the mechanism of porphyrin accumulation after the administration of ALA and to investigate whether ALA-induced protoporphyrin IX might be a suitable photosensitizer in Barrett's oesophagus and adenocarcinoma, we determined the activities of porphobilinogen deaminase (PBG-D) and ferrochelatase (FC) in various malignant and premalignant as well as in normal tissues of the human oesophagus. A PDT power index for ALA-induced porphyrin accumulation, the ratio of PBG-D to FC normalized for the normal squamous epithelium of the oesophagus, was calculated to evaluate intertissue variation in the ability to accumulate porphyrins. In malignant and premalignant tissue a twofold increased PBG-D activity and a marginally increased FC activity was seen compared with normal squamous epithelium. A significantly increased PDT power index in Barrett's epithelium and adenocarcinoma was found. Our results suggest that, after the administration of ALA, porphyrins will accumulate in a greater amount in Barrett's epithelium and adenocarcinoma of the oesophagus because of an imbalance between PBG-D and FC activities. The PDT power index here defined might be a useful indicative parameter for predicting the susceptibility of these tissues to ALA-PDT.


British Journal of Cancer (1998) 78(5), 679–682
© 1998 Cancer Research Campaign

### Biochemical basis of 5-aminolaevulinic acid-induced protoporphyrin IX accumulation: a study in patients with (pre)malignant lesions of the oesophagus

P Hinnen[1], FWM de Rooij[1], MLF van Velthuysen[2], A Edixhoven[1], R van Hillegersberg[3], HW Tilanus[3], JHP Wilson[1] and PD Siersema[1]

Departments of [1]Gastroenterology & Hepatology (Internal Medicine II), L455, [2]Pathology and [3]Surgery, University Hospital Rotterdam-Dijkzigt, Rotterdam, Dr. Molewaterplein 40, 3015 GD Rotterdam, The Netherlands

**Summary** Administration of 5-aminolaevulinic acid (ALA) leads to porphyrin accumulation in malignant and premalignant tissues, and ALA is used as a prodrug in photodynamic therapy (PDT). To understand the mechanism of porphyrin accumulation after the administration of ALA and to investigate whether ALA-induced protoporphyrin IX might be a suitable photosensitizer in Barrett's oesophagus and adenocarcinoma, we determined the activities of porphobilinogen deaminase (PBG-D) and ferrochelatase (FC) in various malignant and premalignant as well as in normal tissues of the human oesophagus. A PDT power index for ALA-induced porphyrin accumulation, the ratio of PBG-D to FC normalized for the normal squamous epithelium of the oesophagus, was calculated to evaluate intertissue variation in the ability to accumulate porphyrins. In malignant and premalignant tissue a twofold increased PBG-D activity and a marginally increased FC activity was seen compared with normal squamous epithelium. A significantly increased PDT power index in Barrett's epithelium and adenocarcinoma was found. Our results suggest that, after the administration of ALA, porphyrins will accumulate in a greater amount in Barrett's epithelium and adenocarcinoma of the oesophagus because of an imbalance between PBG-D and FC activities. The PDT power index here defined might be a useful indicative parameter for predicting the susceptibility of these tissues to ALA-PDT.

**Keywords:** photodynamic therapy; haem biosynthesis; porphyria; Barrett's oesophagus; adenocarcinoma of the oesophagus

Haem biosynthesis (Figure 1) is essential to every cell and requires eight molecules of glycine and eight molecules of succinyl CoA for each molecule of haem. The first intermediate is 5-aminolaevulinic acid (ALA); two molecules of ALA are converted to porphobilinogen, which is metabolized to porphyrinogen intermediates by porphobilinogen deaminase (PBG-D). The last step is the incorporation of iron into protoporphyrin IX (PPIX), catalysed by ferrochelatase (FC). The synthesis of ALA is the rate-limiting step. If exogenous ALA is provided, then other enzymes become rate-limiting in haem formation.

Some cancer cells have been found to have an increased PBG-D activity of (Rubino and Rasetti, 1966; Schoenfeld et al, 1988*a*; Navone et al, 1990, 1991; el-Sharabasy et al, 1992), and in most studies these cells have been found to have a decreased FC activity (Rubino and Rasetti, 1966; Dailey and Smith, 1984; Smith, 1987; el-Sharabasy et al, 1992; Van Hillegersberg et al, 1992). For such cells, administration of ALA will lead to the accumulation of porphyrins, especially PPIX (Anderson et al, 1981). This provides a biological rationale for the clinical use of ALA photodynamic therapy (ALA-PDT).

Barrett's (columnar-lined) oesophagus results from long-term gastro-oesophageal reflux (Mossberg, 1966). It is of clinical importance because of its malignant potential. Barrett's oesophagus can lead to the development of adenocarcinoma through a multistep process of progression from metaplasia to low-grade dysplasia, high-grade dysplasia and ultimately to invasive cancer (Hamilton and Smith, 1987; Hameeteman et al, 1989). High-grade dysplasia in Barrett's oesophagus presents a difficult management problem. Options include endoscopic surveillance and/or oesophagectomy (Levine et al, 1993; Clark et al, 1996; Cameron, 1997). A new non-surgical management option involves eradicating the dysplastic epithelium and columnar mucosa by PDT. In contrast to other photosensitizers, many of which localize in the microvasculature of all tissue layers of hollow organs, ALA induces much higher levels of PPIX in the mucosa than submucosa or muscularis mucosae (Loh et al, 1993). ALA-PDT has been used to treat high-grade dysplasia in Barrett's oesophagus, resulting in necrosis of dysplastic mucosa with regeneration of normal squamous mucosa (Gossner et al, 1995; Regula et al, 1995; Barr et al, 1996).

To optimize ALA-PDT for Barrett's oesophagus and early carcinoma, knowledge of the mechanism of porphyrin accumulation in these tissues is required. We determined the activities of PBG-D and FC in normal tissue as well as in malignant and premalignant tissue of the human oesophagus. These two enzymes play an important role after the administration of ALA: PBG-D is in many cells the rate-limiting enzyme when exogenous ALA is administered and FC is the enzyme directly responsible for the conversion of PPIX to haem. We propose the use of a PDT power index for the intertissue variation in the ability to accumulate PPIX, in order to create a parameter that might indicate the susceptibility of tissues to ALA-PDT.

*Received 28 November 1997*
*Revised 9 March 1998*
*Accepted 11 March 1998*

*Correspondence to:* FWM de Rooij

**Figure 1** Haem biosynthetic pathway

### MATERIALS AND METHODS

### Tissue samples

Between August 1996 and February 1997 tissue was obtained from 27 patients (16 men and 11 women) undergoing an oesophageal resection. The mean age was 61 years (43–81 years). Nine patients had a squamous cell carcinoma, 18 had an adenocarcinoma of the distal oesophagus and in nine of these patients Barrett's epithelium was present. Samples from histologically proven Barrett's mucosa, squamous cell carcinoma and adenocarcinoma as well as samples from normal gastric mucosa and normal squamous epithelium were taken immediately after the resection. In some instances, samples could not be taken from Barrett's mucosa. Tissue samples were embedded in formalin, sectioned and stained with haematoxylin and eosin. The grade of tumour differentiation was described as well as the grade of dysplasia in Barrett's mucosa. Barrett's mucosa was classified as indefinite, low-grade dysplasia (LGD) and high-grade dysplasia (HGD). In addition, adjacent tissue samples were frozen (–70°C) until the moment of biochemical analysis. All determinations were performed in duplicate within 6 weeks of resection. Control experiments showed no change in activities in samples stored at –70°C for this time. This temperature was found to be essential; when stored at –20°C, FC activity decreased within a few days, whereas the PBG-D activity remained stable for weeks.

### Chemicals

The following reagents were obtained from Porphyrin Products (Logan, UT, USA): PPIX disodium salt, zinc PPIX and porphobilinogen (PBG). Coproporphyrin and Triton X-100 were purchased from Sigma Chemical (St Louis, MO, USA). Tris-HCl was purchased from Boehringer Mannheim (Mannheim, Germany) and other chemicals were purchased from Merck (Darmstadt, Germany).

**Table 1** Enzyme activities and PDT power indexes

| | PBG-D[a] | Range | |
|---|---|---|---|
| Squamous epithelium | $n = 27$ | 22.8 ± 7.3 | (10–42) |
| Gastric mucosa | $n = 27$ | 24.9 ± 8.6 | (10–42) |
| Barrett's epithelium | $n = 7$ | 40.6 ± 13.7† | (21–63) |
| Adenocarcinoma | $n = 17$ | 55.0 ± 19.9‡ | (25–93) |
| Squamous cell carcinoma | $n = 9$ | 37.6 ± 14.1† | (21–67) |
| *Ferrochelatase*[a] | | | |
| Squamous epithelium | $n = 24$ | 391 ± 152 | (124–718) |
| Gastric mucosa | $n = 24$ | 685 ± 265‡ | (336–1187) |
| Barrett's epithelium | $n = 6$ | 437 ± 203† | (176–726) |
| Adenocarcinoma | $n = 16$ | 582 ± 220‡ | (230–1048) |
| Squamous cell carcinoma | $n = 7$ | 558 ± 332 | (251–1263) |
| *PDT power index* | | | |
| Squamous epithelium | $n = 24$ | 1.0 | |
| Gastric mucosa | $n = 24$ | 0.7 ± 0.2‡ | (0.3–1.1) |
| Barrett's epithelium | $n = 6$ | 1.8 ± 0.8† | (0.8–3.0) |
| Adenocarcinoma | $n = 16$ | 1.9 ± 1.2‡ | (0.6–5.6) |
| Squamous cell carcinoma | $n = 7$ | 1.1 ± 0.5 | (0.6–2.0) |

[a]pmol mg protein$^{-1}$ h$^{-1}$, mean ± s.d. (†$P < 0.05$, ‡$P < 0.01$).

### FC and PBG-D assays

FC activity was measured by a modification of the method of Li et al (1987) as described previously (Van Hillegersberg et al, 1992). PBG-D measurements were performed by a modification of the method of Wilson et al (1986). Tissue samples, kept on ice, were homogenized in water (1:5 wt:wt) using a Potter Elvehjem homogenizer (Kontess Glass, Vineland, NJ, USA). An aliquot of 50 µL of a 200 M solution of PBG in 0.1 M Tris-HCL buffer, pH 8.0, was added to 50 µl of the homogenate. This mixture was incubated for 1 h at 37°C. The reaction was stopped by adding 600 µl of Tris-HCl buffer (Tris-HCl 50 mM, trichloroacetic acid 1.5 M in aqua dest.) (5:7, v:v). After 5 min exposure to UV light (350 nm), to

 © Cancer Research Campaign 1998

convert porphyrinogens to porphyrins, the samples were centrifuged for 10 min at 14 000 *g* (Eppendorf centrifuge, Merck Nederland, The Netherlands), and the fluorescence of the supernatant was measured at 408 nm excitation and 648 nm emission wavelength (Perkin Elmer LS 5B with a red sensitive photomultiplier). Values were calculated according to a standard curve of coproporphyrin III in Tris-HCl buffer (1:1, v:v). Results were expressed as pmol of porphyrins formed per mg protein per hour. Protein was determined according to the method of Lowry et al (1951).

### The PDT power index

The ratio of PBG-D to FC, introduced as the PDT power index, was calculated, with the enzyme activities in each tissue sample relative to the activities in normal squamous epithelium per individual. This index was calculated according to the formula:

[PBG-D(tissue)/FC(tissue)] ×

[FC(squamous epithelium)/PBG-D (squamous epithelium)]

### Statistical analysis

Data are expressed as means ± s.d. and were analysed for statistical significance using the Wilcoxon matched-pairs signed rank-sum test. The enzyme activities of the malignant and premalignant tissues were compared with the adjacent normal tissue in the same patient.

### RESULTS

All results are shown in Table 1. A twofold increase in PBG-D activity was found in Barrett's epithelium ($P = 0.018$) and in adenocarcinoma of the oesophagus ($P = 0.001$) compared with normal squamous epithelium. Regarding the activity of FC, although the mean values were significantly increased compared with normal squamous epithelium, this increase was less marked compared with the PBG-D activity increase. This resulted in a significant increase in the PDT power index in Barrett's oesophagus ($P = 0.046$) and adenocarcinoma ($P = 0.003$) compared with the normal squamous epithelium. Of the five cases of Barrett's oesophagus in which the index was calculated and the grade of dysplasia determined, four cases were classified as LGD and one case as HGD. The PDT power indexes were 0.8, 1.4, 1.4, 1.6 for LGD and 2.4 for HGD.

### DISCUSSION

Several groups have shown that porphyrins accumulate in neoplastic tissue after the administration of ALA (Peng et al, 1997, review). Normally, haem synthesis is regulated by substrate availability and by feedback inhibition of the enzyme ALA synthase. The concentration of substrates and intermediates are usually far below the Michaelis constants of the enzymes, in which case intermediates are metabolized to haem (Bottomly and Muller-Eberhard, 1988). When exogenous ALA is administered, normal cells will rapidly produce haem. An excess of exogenous ALA will initially overload the system and porphyrin intermediates will accumulate. The presence of the intermediates contributes to photosensitivity of normal cells, but these intermediates are rapidly metabolized into haem. In malignant and premalignant tissue of the oesophagus, we found increased PBG-D and FC activities compared with normal squamous epithelium, and an imbalance between these activities. These results are in line with those found in our previous smaller studies (Hinnen et al, 1997*a*, b). Owing to individual patient and tissue variations in the activities of PBG-D and FC, also described by others (Rubino and Rasetti, 1966; Dailey and Smith, 1984; Schoenfeld et al, 1987, 1988*a,b*; Smith, 1987; Navone et al, 1990; el-Sharabasy et al, 1992; Van Hillegersberg et al, 1992), the activity of these two haem enzymes can be better described relative to each other. This ratio, which we propose to call the PDT power index, reflects the enzymatic ability of cells to accumulate porphyrins after ALA administration and might predict the susceptibility of tissue to ALA-PDT. The PDT power index was significantly increased in Barrett's epithelium and adenocarcinoma compared with normal squamous epithelium, indicating that the FC activity was relatively low compared with the PBG-D activity. As this index can also be derived from biopsy specimens, e.g. oesophagus or Barrett, it could be applied before ALA-PDT to estimate tissue susceptibility.

Increased PBG-D activity relative to normal tissue has consistently been found in tumours (Schoenfeld et al, 1988*a*; Navone et al, 1990, 1991; el-Sharabasy et al, 1992) as well as in rapidly dividing cells, e.g. regenerating liver cells (Schoenfeld et al, 1987, 1988*b*), suggesting that this phenomenon might be common in situations of increased cell replication. In contrast to the consistent studies concerning the activity of PBG-D, there seems to be a difference in FC activity among different tumour types (Rubino and Rasetti, 1966; Dailey and Smith, 1984; Smith, 1987; el-Sharabasy et al, 1992; Van Hillegersberg et al, 1992). Dailey and Smith (1984) found a decreased FC activity in the Morris hepatoma model; however, they also pointed out that some porphyrins can act as inhibitors of FC. Smith (1987) found decreased FC activities in human skin tumours but also in normal skin tissue compared with those in rat liver mitochondria. El-Sharabasy et al (1992) determined the FC activity in whole-blood samples of children and adults with acute lymphoblastic leukaemia (ALL), non-Hodgkin's lymphoma (NHL) or Hodgkin's disease (HD) and found lowered activities in patients with ALL, slightly decreased activities and increased activities of FC in blood of patients with NHL and HD respectively compared with healthy control groups. Compared with liver, which is one of the main haem-synthesizing tissues, most tissues have low enzyme activities (Webber et al, 1997). Our group found the FC activity to be decreased in a colon carcinoma liver metastasis model and we suggested applying ALA-PDT to patients with these liver metastases (Van Hillegersberg et al, 1992). Regarding the effect of the storage temperature on FC activity, interpretation of data from other studies might be biased because of differences in tissue storage.

In the gastrointestinal tract, accumulation of porphyrins after ALA administration is more pronounced in the mucosa than in the underlying submucosa and muscle layers, making ALA suitable for treating most mucosal lesions (Bedwell et al, 1992; Loh et al, 1993; Webber et al, 1997). In patients with familial adenomatous polyposis, no differences in PPIX concentrations were found between normal and adenomatous tissue (Mlkvy et al, 1995). In the DMH rat colonic tumour model the same group showed differences in the levels of PPIX between normal mucosa and tumour with a ratio of 1:6 (Bedwell et al, 1992). In another study they showed that higher doses of ALA (60 mg $kg^{-1}$ instead of 30 mg $kg^{-1}$) improved the tumour–normal mucosa PPIX sensitization ratio in patients with

© *Cancer Research Campaign 1998*

colon carcinoma (Regula et al. 1995). Webber et al (1997) showed selective accumulation of PPIX in adenocarcinomas of the gastrointestinal tract in 42 patients. Our biochemical study has characterized the enzymatic capacities of haem biosynthesis in normal, premalignant and malignant tissue of the human oesophagus. These results provide evidence for the selectivity of PPIX accumulation between normal and neoplastic tissue of the oesophagus. Whether selective PPIX accumulation creates the possibility of achieving selective necrosis is still in question. Recently, Bown and Millson (1997) have stated that the selectivity of ALA-PDT-induced necrosis, in the gastrointestinal tract, is between mucosa and underlying submucosa and muscularis and not between normal mucosa and neoplastic mucosa.

In conclusion, our study supports the use of ALA for selective PDT in Barrett's oesophagus and early carcinoma. Information about the PDT power index could be useful in predicting the effect of ALA administration on porphyrin accumulation and therefore on the susceptibility of the disorder to ALA-PDT.

### ACKNOWLEDGEMENTS

This work was supported by a grant from ASTRA Pharmaceutica, The Netherlands.

### REFERENCES

Anderson KE, Drummond GS, Freddara U, Sardana MK and Sassa S (1981) Porphyrogenic effects and induction of heme oxygenase in vivo by delta-aminolevulinic acid. *Biochim Biophys Acta* **676**: 289–299

Barr H, Shepherd NA, Dix A, Roberts DJ, Tan WC and Krasner N (1996) Eradication of high-grade dysplasia in columnar-lined (Barrett's) oesophagus by photodynamic therapy with endogenously generated protoporphyrin IX. *Lancet* **348**: 584–585

Bedwell J, MacRobert AJ, Phillips D and Bown SG (1992) Fluorescence distribution and photodynamic effect of ALA-induced PP IX in the DMH rat colonic tumour model. *Br J Cancer* **65**: 818–824

Bottomly SS and Muller-Eberhard U (1988) Pathophysiology of heme synthesis. *Semin Hematol* **25**: 282–302

Bown S and Millson C (1997) Photodynamic therapy in gastroenterology. *Gut* **41**: 5–7

Cameron AJ (1997) Barrett's esophagus: does the incidence of adenocarcinoma matter? *Am J Gastroenterol* **92**: 193–194

Clark GW, Ireland AP and DeMeester TR (1996) Dysplasia in Barrett's esophagus: diagnosis, surveillance and treatment. *Dig Dis* **14**: 213–227

Dailey HA and Smith A (1984) Differential interaction of porphyrins used in photoradiation therapy with ferrochelatase. *Biochem J* **223**: 441–445

el-Sharabasy MM, el-Waseef AM, Hafez MM and Salim SA (1992) Porphyrin metabolism in some malignant diseases. *Br J Cancer* **65**: 409–412

Gossner L, Sroka R, Hahn EG and Ell C (1995) Photodynamic therapy: successful destruction of gastrointestinal cancer after oral administration of aminolevulinic acid. *Gastrointest Endosc* **41**: 55–58

Hameeteman W, Tytgat GN, Houthoff HJ and van den Tweel JG (1989) Barrett's esophagus: development of dysplasia and adenocarcinoma. *Gastroenterology* **96**: 1249–1256

Hamilton SR and Smith RR (1987) The relationship between columnar epithelial dysplasia and invasive adenocarcinoma arising in Barrett's esophagus. *Am J Clin Pathol* **87**: 301–312

Hinnen P, de Rooij FWM, van Velthuysen MLF, Edixhoven-Bosdijk A, Tilanus HW, Wilson JHP and Siersema PD (1997*a*) An imbalance between haem biosynthetic enzymes results in an increased photodynamic therapy power index in (pre)malignant tissue of the esophagus. *Acta Haemato* **98**: A407

Hinnen P, de Rooij FWM, van Velthuysen MLF, Edixhoven-Bosdijk A, Tilanus HW, Wilson JHP and Siersema PD (1997*b*) Increased photodynamic therapy power index in (pre)malignant tissue of the oesophagus. *Endoscopy* **29**: E9

Levine DS, Haggitt RC, Blount PL, Rabinovitch PS, Rusch VW and Reid BJ (1993) An endoscopic biopsy protocol can differentiate high-grade dysplasia from early adenocarcinoma in Barrett's esophagus. *Gastroenterology* **105**: 40–50

Li F, Lim CK and Peter TJ (1987) An HPLC assay for rat liver ferrochelatase activity. *Biomed Chromatogr* **2**: 164–168

Loh CS, Vernon D, MacRobert AJ, Bedwell J, Bown SG and Brown SB (1993) Endogenous porphyrin distribution induced by 5-aminolaevulinic acid in the tissue layers of the gastrointestinal tract. *J Photochem Photobiol B* **20**: 47–54

Lowry O, Rosebrough N, Farr A and Randall R (1951) Protein measurement with the Folin phenol reagent. *J Biol Chem* **193**: 265–275

Mlkvy P, Messmann H, Debinski H, Regula J, Conio M, MacRobert A, Spigelman A, Phillips R and Bown SG (1995) Photodynamic therapy for polyps in familial adenomatous polyposis – a pilot study. *Eur J Cancer* **31A**: 1160–1165

Mossberg SM (1966) The columnar-lined esophagus (Barrett syndrome) – an acquired condition? *Gastroenterology* **50**: 671–676

Navone NM, Polo CF, Frisardi AL, Andrade NE and Battle AM (1990) Heme biosynthesis in human breast cancer – mimetic 'in vitro' studies and some heme enzymic activity levels. *Int J Biochem* **22**: 1407–1411

Navone NM, Polo CF, Frisardi AL and Battle AM (1991) Mouse mammary carcinoma porphobilinogenase and hydroxymethyl bilane synthetase. *Comparat Biochem Physiol* **98B**: 67–74.

Peng Q, Warloe T, Berg K, Moan J, Kongshaug M, Giercksky KE and Nesland JM (1997) 5-Aminolevulinic acid-based photodynamic therapy. Clinical research and future challenges. *Cancer* **79**: 2282–2308

Regula J, MacRobert AJ, Gorchein A, Buonaccorsi GA, Thorpe SM, Spencer GM, Hatfield AR and Bown SG (1995) Photosensitisation and photodynamic therapy of oesophageal, duodenal, and colorectal tumours using 5 aminolaevulinic acid induced protoporphyrin IX – a pilot study. *Gut* **36**: 67–75

Rubino GF and Rasetti L (1966) Porphyrin metabolism in human neoplastic tissues. *Panminerva Med* **8**: 290–292

Schoenfeld N, Mamet R, Epstein O, Lahav M, Lurie Y and Atsmon A (1987) The heme biosynthetic pathway in the regenerating rat liver. The relation between enzymes of heme synthesis and growth. *Eur J Biochem* **166**: 663–666

Schoenfeld N, Epstein O, Lahav M, Mamet R, Shaklai M and Atsmon A (1988*a*) The heme biosynthetic pathway in lymphocytes of patients with malignant lymphoproliferative disorders. *Cancer Lett* **43**: 43–48

Schoenfeld N, Mamet R, Leibovici L, Epstein O, Teitz Y and Atsmon A (1988*b*) Growth rate determines activity of porphobilinogen deaminase both in nonmalignant and malignant cell lines. *Biochem Med Metab Biol* **40**: 213–217

Smith A (1987) Mechanisms of toxicity of photoactivated artificial porphyrins. Role of porphyrin–protein interactions. *Ann N Y Acad Sci* **514**: 309–322

Van Hillegersberg R, Van den Berg JW, Kort WJ, Terpstra OT and Wilson JH (1992) Selective accumulation of endogenously produced porphyrins in a liver metastasis model in rats. *Gastroenterology* **103**: 647–651

Webber J, Kessel D and Fromm D (1997) Side effects and photosensitization of human tissues after aminolevulinic acid. *J Surg Res* **68**: 31–37

Wilson JHP, de Rooij FWM and te Velde K (1986) Acute intermittent porphyria in The Netherlands. *Neth J Med* **29**: 393–399

© Cancer Research Campaign 1998